# Live-cell imaging of septins and cell polarity proteins in the growing dikaryotic vegetative hypha of the model mushroom *Coprinopsis cinerea*

**DOI:** 10.1038/s41598-023-37115-y

**Published:** 2023-06-22

**Authors:** Tetsuya Kakizaki, Haruki Abe, Yuuka Kotouge, Mitsuki Matsubuchi, Mayu Sugou, Chiharu Honma, Kouki Tsukuta, Souichi Satoh, Tatsuhiro Shioya, Hiroe Nakamura, Kevin S. Cannon, Benjamin L. Woods, Amy Gladfelter, Norio Takeshita, Hajime Muraguchi

**Affiliations:** 1 Department of Biotechnology, Faculty of Bioresource Sciences, Akita Prefectural University, Shimoshinjo-nakano, Akita, 010-0195 Japan; 2 Department of Biology, University of North Carolina at Chapel Hill, Chapel Hill, NC USA; 3 Microbiology Research Center for Sustainability (MiCS), Faculty of Life and Environmental Sciences, University of Tsukuba, Tennodai 1-1-1, Tsukuba, 305-8572 Japan; 4 Present Address: Department of Cell Biology, Duke University, Durham, USA

**Keywords:** Fungal biology, Cellular imaging

## Abstract

The developmental biology underlying the morphogenesis of mushrooms remains poorly understood despite the essential role of fungi in the terrestrial environment and global carbon cycle. The mushroom *Coprinopsis cinerea* is a leading model system for the molecular and cellular basis of fungal morphogenesis. The dikaryotic vegetative hyphae of this fungus grow by tip growth with clamp cell formation, conjugate nuclear division, septation, subapical peg formation, and fusion of the clamp cell to the peg. Studying these processes provides many opportunities to gain insights into fungal cell morphogenesis. Here, we report the dynamics of five septins, as well as the regulators CcCla4, CcSpa2, and F-actin, visualized by tagging with fluorescent proteins, EGFP, PA-GFP or mCherry, in the growing dikaryotic vegetative hyphae. We also observed the nuclei using tagged Sumo proteins and histone H1. The five septins colocalized at the hyphal tip in the shape of a dome with a hole (DwH). CcSpa2-EGFP signals were observed in the hole, while CcCla4 signals were observed as the fluctuating dome at the hyphal tip. Before septation, CcCla4-EGFP was also occasionally recruited transiently around the future septum site. Fluorescent protein-tagged septins and F-actin together formed a contractile ring at the septum site. These distinct specialized growth machineries at different sites of dikaryotic vegetative hyphae provide a foundation to explore the differentiation program of various types of cells required for fruiting body formation.

## Introduction

*Coprinopsis cinerea* is a model mushroom and grows vegetatively as typical dikaryotic hyphae, which usually produce fruiting bodies consisting of an obvious cap and stipe^1–3^. Fruiting body formation begins with the aggregation of aerial vegetative hyphae, producing hyphal knots of approximately 0.2 mm or less in diameter. In the hyphal knots, cells divide rapidly and differentiate into a compact core composed of highly branched short cells and a layer of veil cells covering the core^4^. Following differentiation of the primordial shaft, the rudimentary pileus (cap) differentiates at the upper region of the primordial shaft, forming a tiny fruiting body primordium^1^. The fruiting body is composed of cells that exhibit different growth modes: hyphae growing at the hyphal tip, cells that rapidly divide without directional polarized growth, and cells displaying diffuse extension growth, which is characterized by expansion of the overall cell surface. Stipe and veil cells exhibit typical diffuse extension growth and provide an opportunity to study fungal cell elongation^5,6^.

While there is limited understanding of the molecular basis of mushroom body assembly in any system, the septin family of proteins have been linked to the process. A defect in the promoter region of a septin gene of *C. cinerea*, *Cccdc3*, resulted in a failure of stipe cell elongation at the maturation stage of fruiting^7^. Septins were originally identified as *cdc* (cell division cycle) mutants in *Saccharomyces cerevisiae*^8^. They are GTP-binding proteins conserved from fungi to mammals and some algae species^9^, and form heteromeric complexes that assemble into filaments^10^ and various higher-order structures in cells^11,12^. Since septins have a polybasic domain involved in membrane binding^13^, various higher-order structures of septins are frequently associated with the cell cortex. Subcellular localization of septins has been observed and investigated in yeasts, suggesting that septins play roles in recruitment of cytokinetic proteins, morphogenesis checkpoint, and membrane curvature sensing^11,14,15^. The subcellular localizations of septins have also been observed in a variety of filamentous fungi^16–20^.

Consistent with CcCdc3 being essential for stipe cell elongation, EGFP-tagged CcCdc3 septin assembles into abundant thin filaments at the cortex of stipe cells^7^. Notably, the EGFP-CcCdc3 signals were observed in the stipe cells and the vegetative hyphae, suggesting that CcCdc3 also functions in vegetative hyphal growth. The *C. cinerea* genome contains at least 5 septin genes encoding CcCdc3, CcCdc10, CcCdc11a, CcCdc11b and CcCdc12^21^. CcCdc11a and CcCdc11b are included in a clade of Shs1/Sep7 septin found in *S. cerevisiae*^7^. Expression of the septin genes except for *Cccdc11a* are upregulated during stipe elongation^7,22^.

A variety of proteins have been implicated in septin organization of *S. cerevisiae*^23,24^*.* The Cla4 kinase has been shown to have synthetic lethal interactions with various genes, which encode a septin and proteins involved in cell polarity and cell wall synthesis^25,26^, and has been demonstrated to interact directly with and phosphorylate certain septins^24^. The Cla4 kinase activity has been reported to be regulated by the Rho family GTPase Cdc42^27^, which has also been implicated in the recruitment of the yeast septins^28^ and regulation of the polarisome including Spa2^23,29^. In various filamentous fungi, Cla4 functions have also been implicated in cell morphogenesis^30–34^. Septins in *S. cerevisiae* have been reported to be modified not only by phosphorylation but also by sumoylation^35–37^.

Growth of the dikaryotic vegetative hyphae in model mushrooms includes various cellular events and provides many opportunities to analyze fungal cell morphogenesis^38–40^. Tip growth is observed in the hyphal tip and the tip of the clamp cell, which is formed as a protrusion from the preexisting hyphal cell wall under which the two conjugate nuclei overlap and determine to divide^38^. In *C. cinerea*, the leading nucleus of the conjugate nuclei enters the clamp cell and divides. The clamp cell fuses to the subapical cell to release the nucleus trapped in the clamp cell into the main hypha. Before the clamp cell fuses to the subapical cell, the subapical cell responds to form a subapical peg^41^.

Here, we report the dynamics of fluorescent protein-tagged septins, CcCla4, CcSpa2, two CcSumo proteins and Lifeact peptide, as well as histone H1 in the dikaryotic vegetative hyphae of *C. cinerea*. Live cell imaging allows us to observe the transient assembly of tagged proteins to specific sites at a specific time in cell cycle progression. Two color tagging of proteins in interest provides insights into relationships between septins and the related proteins in hyphal tip growth, clamp cell formation, septum formation, and subapical peg formation. These studies delineate an atlas of key molecular assemblies for a major model mushroom, providing a foundation for molecular understanding of the developmental biology of mushrooms.

## Results

### Dynamics of septins tagged with fluorescent proteins at the hyphal tip

The *C. cinerea* septin CcCdc3 tagged with EGFP localized at the cortex of stipe cells and in dikaryotic vegetative hyphae^7^. The *C. cinerea* has at least five septins, CcCdc3, CcCdc10, CcCdc11a, CcCdc11b and CcCdc12. Septins often form a complex with each other and assemble into non-polar filaments^10^. However, there are also examples of septins acting independently of a complex such as with AspE or SEPT9^42,43^. To examine the degree to which septins colocalize at certain time points or places in *C. cinerea*, two different septins were tagged with different color fluorescent proteins, EGFP or mCherry, and observed in the growing dikaryotic vegetative hyphae with a fluorescence microscope to analyze through time.

**Figure 1:**
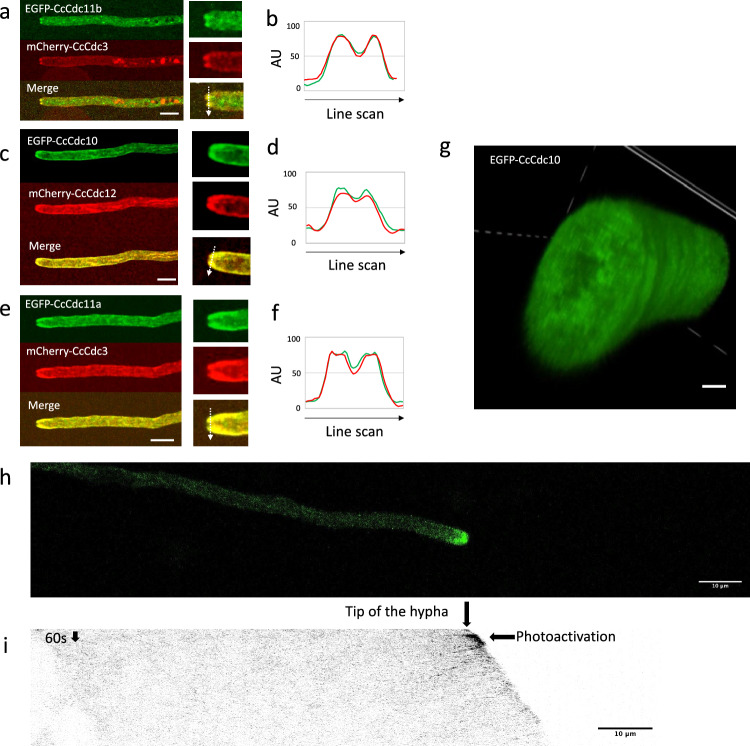
Localization of septins in the tip region of the dikaryotic vegetative hypha. (**a**) EGFP-CcCdc11b and mCherry-CcCdc3 localizations at the growing hyphal tip. The right panels indicate the enlarged tip. See Supplementary Movie 1. (**b**) Line scan analysis along an arrow in the enlarged merged image in panel (**a**). (**c**) EGFP-CcCdc10 and mCherry-CcCdc12 localizations at the growing hyphal tip. The right panels indicate the enlarged tip. See Supplementary Movie 2. (**d**) Line scan analysis along an arrow in the enlarged merged image in panel (**c**). (**e**) EGFP-CcCdc11a and mCherry-CcCdc3 localizations at the growing hyphal tip. The right panels indicate the enlarged tip. See Supplementary Movie 3. (**f**) Line scan analysis along an arrow in the merged image in panel (**e**). (**g**) 3D image of the growing apical cell expressing EGFP-CcCdc10, showing a septin dome with a hole (DwH) at the hyphal tip. Scale bar: 1 µm. See Supplementary Movie 4 (Z stack slice step: 0.2 µm). (**h**) PA-GFP-CcCdc3 showing a backward movement of CcCdc3 from the hyphal tip after photoactivation. Scale bar: 10 µm. See Supplementary Movie 5. (**i**) Kymograph of panel h along a line placed at the center of the hypha. Scale bar: 10 µm.

The five septins tagged with fluorescent proteins accumulated as two spots near the hyphal tip. They did not occupy the very tip of the growing hypha, suggesting that septins form a ring at the hyphal tip region or are excluded in some way from the very apex (Fig. 1; Supplementary Movies 1–4). The fluorescent signals from the tags fused to CcCdc3, CcCdc10, CcCdc11a and CcCdc12 were observed to expand from the septin ring towards the subapical region on the plasma membrane, resulting in a septin dome with a hole (DwH) at the growing hyphal tip region.

In contrast, EGFP-CcCdc11b signals appeared to display somewhat different behaviors than other tagged septins. EGFP-CcCdc11b colocalized with other tagged septins as the ring at the hyphal tip, at the basal part of the protruding clamp cell and branch and at the septum. However, the cortical localization of EGFP-CcCdc11b was reduced compared with other cortical septins (Fig. 1a,c,e). In Supplementary Movies 1–3, the nuclei were observed as relatively large dark areas and EGFP-CcCdc11b signals were observed as puncta in the front of the nuclei. The structures with EGFP-CcCdc11b signals dynamically changed in shape to tubular particles. These tubular particles were observed more in the posterior region of the two nuclei (Supplementary Movie 1).

To confirm the dynamic movement of cortical septins, we replaced EGFP with PA-GFP and observed it after photo-activation (Fig. 1h; Supplementary Movie 5). PA-GFP-CcCdc3 signals were observed on the membrane at the hyphal tip region after the photo-activation of the hyphal tip, and the intensity of the signals diminished over time. While the diminished signals would be due, at least in part, to photobleaching or exchange, the PA-GFP signal was observed as a particle moving backward after 2–3 min from the photo-activation (Supplementary Movie 5). Kymograph shows that the hypha can grow continuously before and after the photo-activation and the PA-GFP-CcCdc3 signal moves backward after 2–3 min from the photo-activation from the tip after the photo-activation (Fig. 1i).

### Dynamics of the septin-related cell polarity proteins at the hyphal tip

**Figure 2:**
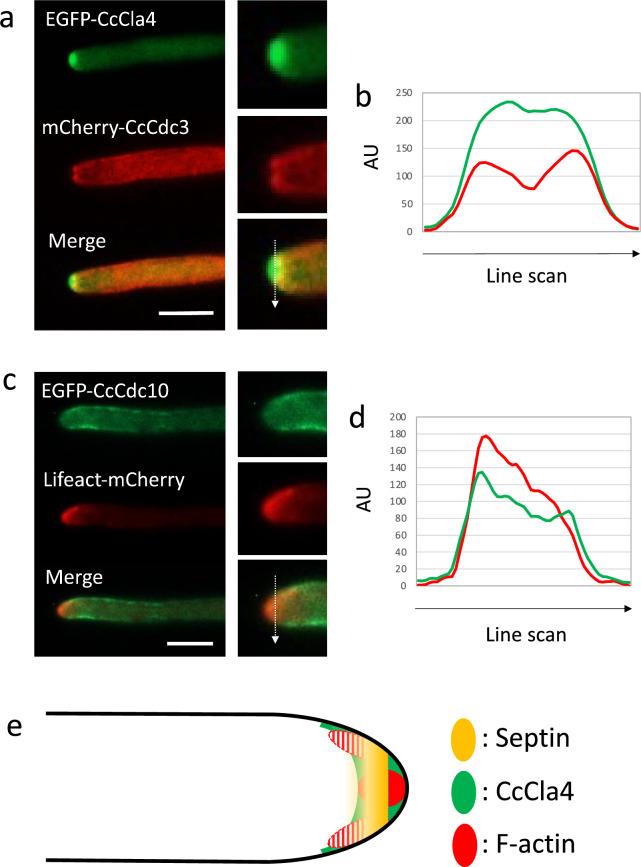
Localization of septin-related cell polarity proteins in the tip region of the dikaryotic vegetative hypha. (**a**) CcCla4-EGFP and mCherry-CcCdc3 localizations at the growing hyphal tip. The right panels indicate the enlarged tip. See Supplementary Movie 6. Localization of CcCla4-EGFP at the growing hyphal tip was also observed in Supplementary Movie 7. (**b**) Line scan analysis along an arrow in the enlarged merged image in panel (**a**). (**c**) EGFP-CcCdc10 and Lifeact-mCherry localizations at the growing hyphal tip. See Supplementary Movie 8. (**d**) Line scan analysis along an arrow in the enlarged merged image in panel (**c**). (**e**) A schematic diagram showing the septin DwH and localizations of CcCla4-EGFP and Lifeact-mCherry at the hyphal tip region. The fluctuated actin patches in the subapical region are shown by red-striped areas surrounded by a dashed line. *AU* arbitrary units. Scale bar: 10 µm.

Septin behaviors in *S. cerevisiae* have been found to be regulated by a variety of proteins^23^. The Cla4 kinase has been implicated in regulating septin organization in *S. cerevisiae*^26,44,45^. To examine the relationship between CcCla4 and Cc-septins (*C. cinerea* septins), CcCla4 was tagged with EGFP. CcCla4-EGFP signals were observed as a fluctuating dome at the hyphal tip. The posterior edge of the CcCla4 dome appeared to overlap with the septin DwH at the subapical region (Fig. 2a,b; Supplementary Movies 6 and 7). The overlapping of their localizations indicates that CcCla4 may function with and/or regulate Cc-septins at the growing hyphal tip.

To observe dynamics of the F-actin in a growing dikaryotic vegetative hypha of *C. cinerea*, the mCherry-tagged Lifeact peptide was expressed by the actin promoter. At the growing hypha in *Aspergillus nidulans*, a subapical endocytic ring has been observed, at which actin/actin binding protein A (ABPA) was localized as endocytic patches^46^. The actin distributions have been observed and investigated in many fungal species, including *C. cinerea* using antibodies^40^, *S. commune*, *N. crassa and A. nidulans* using Lifeact peptide^38,47–49^. At the hyphal tip in *C. cinerea*, Lifeact-mCherry signals were observed as patches, which appeared to be classified into two types: one that occupied the apex of the growing hypha and the other that localized and fluctuated at the subapical region (Fig. 2c; Supplementary Movie 8). The fluorescent protein-tagged septins were observed as a ring at the subapical region: within 1 μm behind the apex of the hypha (Fig. 1). At the subapical region, Lifeact-mCherry signals were observed at the inner side of EGFP-CcCdc10 signals on the plasma membrane (Fig. 2d). The colocalizations of Lifeact-mCherry and EGFP-CcCdc10 signals suggested that F-actin and septins are coordinately assembled in the subapical region and could function there in maintenance of the apical compartment (summarized in Fig. 2e).

**Figure 3:**
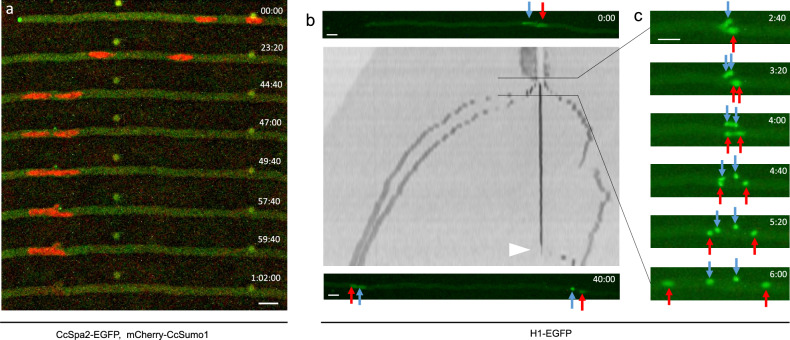
Localization of the polarisome and conjugate nuclear division. (**a**) The growing hypha expressing CcSpa2-EGFP and mCherry-CcSumo1. mCherry-CcSumo1 was accumulated in the nucleus. At time point 00:00, CcSpa2-EGFP signals were observed at the growing hyphal tip. At time point 44:40, the posterior nucleus approached to the leading nucleus. From time points 47:00 to 57:40, CcSpa2-EGFP signals were observed at the tip of the clamp cell growing backward. At time point 57:40, the leading nucleus began to enter the clamp cell. At time point 59:40, CcSpa2-EGFP signals disappeared from the tip of the clamp cell. At time point 1:02:00, mCherry-CcSumo1 signals disappeared from the nuclei during mitosis. See Supplementary Movie 9. (**b**,**c**) The nuclear dynamics in mitosis was observed by tagging histone H1 with EGFP. The anterior and posterior nuclei before mitosis are indicated by blue and red arrows, respectively. In panel (**b**), a kymograph for 40 min between the top and bottom photos is shown. A white arrowhead in the kymograph indicates the point of the release of the nucleus from the clamp cell to the subapical cell. In panel c, the nuclear behavior is shown every 40 s. The anterior nucleus entered the clamp cell, in which the nucleus divided. The posterior nucleus divided into two daughter nuclei in the main hyphal cell. The anterior daughter nucleus occurring in the main hypha passed through the nucleus returned from the clamp cell to the hyphal cell and ran toward the hyphal tip. Scale bar: 10 µm. See Supplementary Movies 10, 11 and schematic diagrams of the nuclear behavior in Fig. 6.

The polarisome has been observed at the apex of the growing hypha in many filamentous fungi and has been considered to nucleate F-actin at the hyphal tip: *Ashbya gossypii*^50^, *Candida albicans*^51^, *Aspergillus nidulans*^52^, *Ustilago maydis*^53^, and *Neurospora crassa*^54^. To observe the polarisome in *C. cinerea*, CcSpa2 was tagged with EGFP. CcSpa2-EGFP signals were observed constitutively as a spherical body at the growing hyphal tip. The rim of the polarisome appeared to overlap with the septin DwH (Fig. 3a; Supplementary Movie 9).

### Clamp formation and conjugate nuclear division

Dikaryotic hyphae of some species in basidiomycetes have clamp connections between cells. A clamp cell is formed to give a space where one nucleus divides during conjugate nuclear division. In Supplementary Movies 1–3, in which the septins were tagged with EGFP or mCherry, the shape of the nucleus was observed as dark area, suggesting that septins did not enter the nucleus. The dark area disappeared during mitosis, suggesting that the nuclear envelope opened.

In *S. cerevisiae*, sumoylation of some septins have been reported^36^. To examine sumoylation of Cc-septins, two sumo proteins, CcSumo1 and CcSumo2, were tagged with mCherry. The mCherry signals from tagged Sumo proteins were not observed in the region where Cc-septins were accumulated, suggesting that most Cc-septins were not sumoylated or that tag interfered with their linkage to the septins. The mCherry signals from tagged CcSumo1 and CcSumo2 were observed only in the interphase nucleus (Fig. 3a; Supplementary Movies 9 and 10) and disappeared during mitosis, consistent with the reports of localizations of sumoylated proteins in *A. nidulans*^55^. When the clamp protruded from the preexisting cell wall, CcSpa2-EGFP signals emerged at the tip of the protrusion (Fig. 3a, time point: 47:00). CcSpa2-EGFP signals stayed at the tip of the growing clamp cell. The leading nucleus visualized by mCherry-CcSumo1 started to enter the growing clamp cell whose tip had CcSpa2-EGFP signals (Fig. 3a, time point: 57:40). During the nuclear entry into the clamp cell, mCherry-CcSumo1 signals disappeared (Fig. 3a, time point: 1:02:00).

To observe the nuclear behavior during mitosis, histone H1 was tagged with EGFP. The leading nucleus entered the clamp cell, in which the nucleus divided (Fig. 3c; Supplementary Movies 10 and 11). After mitosis in the clamp cell, the new anterior nucleus returned to the apical cell and the new posterior nucleus remained trapped in the clamp cell. The nucleus returning from the clamp cell to the apical cell was overtaken by the new anterior sister nucleus in the main hypha. This nuclear behavior is consistent with a previous report in *C. cinerea*^56^. About thirty minutes after trapping the nucleus in the clamp cell, the clamp cell fused to the subapical cell to release the trapped nucleus into the subapical cell, leading to the two nuclei in the subapical cell (Fig. 3b, a white arrowhead).

**Figure 4:**
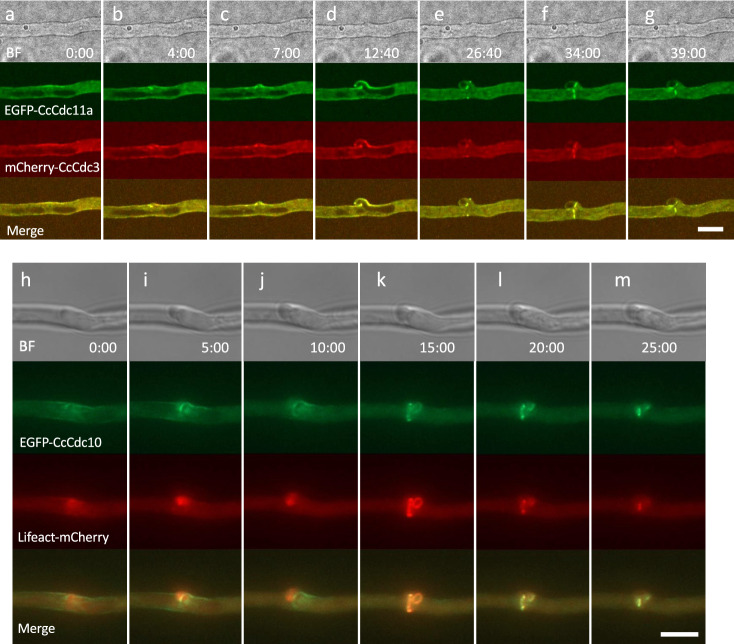
EGFP-CcCdc11a, mCherry-CcCdc3, EGFP-CcCdc10 and Lifeact-mCherry dynamics during clamp formation and septation. (**a**–**g**) Dynamics of EGFP-CcCdc11a and mCherry-CcCdc3 during clamp formation and septation. (**a**) EGFP-CcCdc11a and mCherry-CcCdc3 signals were observed at the cell cortex around the approached nuclei. (**b**) Both septins began to assemble at the one side of the hyphal tube between the two conjugate nuclei. (**b**,**c**) The cell wall began to protrude to form the clamp cell. Both septins are assembled at the basal part of the protrusion. (**d**) Both septins are assembled at the tip of the clamp cell. (**e**) mCherry-CcCdc3 disappeared from the basal part and the surface of the clamp cell. And both septins emerged as spots, which indicate a cross section of a ring, both at the main hypha and the clamp cell to separate the nucleus after mitosis. (**e**–**g**) The diameters of septin rings became narrower. (**g**) The EGFP-CcCdc11a and mCherry-CcCdc3 signals were observed at the septal pore and the subapical peg. See Supplementary Movie 3. (**h**–**m**) The clamp region of the hypha expressing EGFP-CcCdc10 and Lifeact-mCherry. (**h**–**j**) EGFP-CcCdc10 signals became observed around the protrusion of the clamp cell and Lifeact-mCherry signals became localized at the tip of the protrusion. (**k**) After clamp cell formation, EGFP-CcCdc10 and Lifeact-mCherry signals were observed as two rings: one formed in the main hypha and the other at the clamp cell’s anterior side. (**k**–**m**) The diameters of septin rings became narrower. See Supplementary Movie 12 and schematic diagrams of clamp cell formation in Fig. 6. Scale bar: 10 μm.

**Figure 5:**
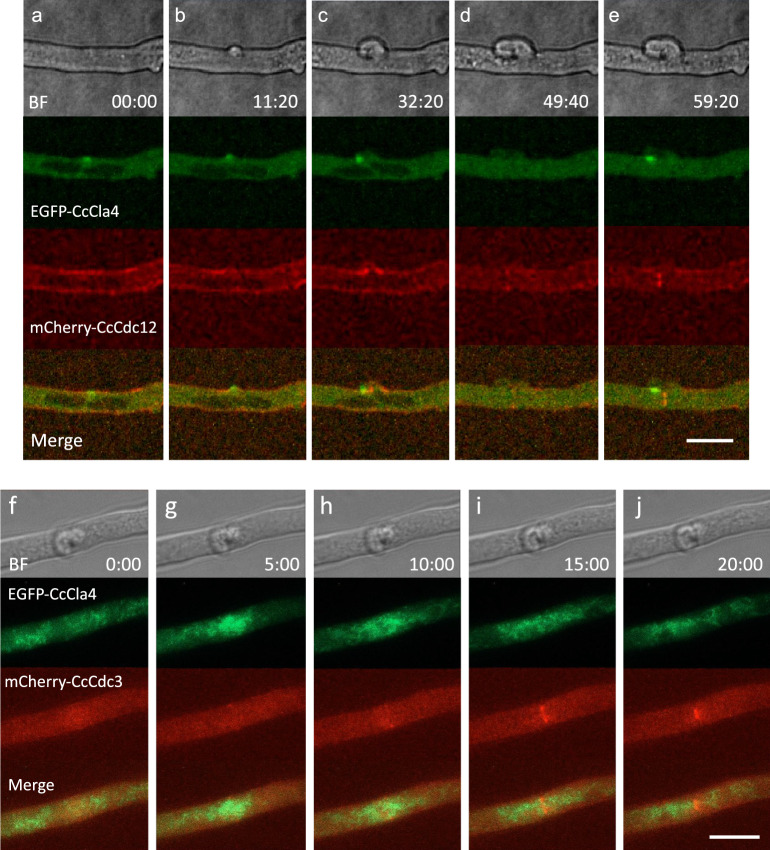
Dynamics of CcCla4-EGFP kinase, mCherry-tagged CcCdc12 and CcCdc3 septins from clamp formation to septation. (**a**–**e**) Dynamics of CcCla4-EGFP and mCherry-CcCdc12 from clamp formation to septation. (**a**) CcCla4-EGFP signals emerged as a spot at the plasma membrane between two conjugate nuclei. (**b**) The site where CcCla4-EGFP assembled began to protrude. (**c**) CcCla4-EGFP signals continued to exist at the tip of the growing clamp cell. mCherry-CcCdc12 signals were observed at the basal part of the clamp cell protrusion. (**d**) CcCla4-EGFP signals disappeared and mCherry-CcCdc12 signals were observed as a ring from which septum formation occurs. (**e**) After the tip of the clamp cell attached to the subapical cell, CcCla4-EGFP signals emerged at the subapical peg site, where mCherry-CcCdc12 signals also assembled. The diameter of the ring visualized by mCherry-CcCdc12 became narrower. See Supplementary Movie 13. (**f**–**j**) Dynamics of CcCla4-EGFP and mCherry-CcCdc3 from clamp formation to septation. After clamp cell formation, CcCla4-EGFP assembled over the broad region around the clamp cell. The assembly of CcCla4-EGFP before septum formation was also observed in Supplementary Movie 14. (**i**–**j**) As CcCla4-EGFP signals dispersed, mCherry-CcCdc3 signals became assembled as a ring to form the septum. (**j**) The diameter of the septin ring visualized by mCherry-CcCdc3 became narrower. Scale bar: 10 μm. See schematic diagrams of clamp cell formation in Fig. 6.

The clamp protrusion started between the two conjugate nuclei or at the site near the anterior edge of the posterior nucleus. The fluorescent signals of the tagged septins were observed around the protrusion of the clamp cell (Fig. 4a–c,h–j; Supplementary Movies 2, 3 and 12). As the clamp cell protruded, the fluorescent signals of the tagged septins were accumulated at the basal part of the protrusion and the tip of the clamp cell (Fig. 4d). During clamp cell elongation, the fluorescent signals of the tagged septins stayed on the cell cortex (Fig. 4d). However, as the septin-actin ring was formed for septum formation (see the next section), these cortical septins disappeared (Fig. 4e–g). CcSpa2-EGFP signals emerged at the tip of the clamp cell (Fig. 3a, time point 47:00–57:40). At the early stage of the clamp protrusion, CcCla4-EGFP signals emerged at the tip of the protrusion (Fig. 5a). The CcCla4-EGFP signals stayed at the tip of the clamp cell growing backward (Fig. 5b,c). The clamp cell protrusion can be considered as the emergence of a new hyphal tip linked to the conjugate nuclear division cycle.

### Septum formation

When the clamp cell protrusion and the conjugate nuclear division were completed, CcCla4-EGFP signals were observed occasionally around the future septum site (Fig. 5g; Supplementary Movie 14). Immediately after the transient assembly of CcCla4-EGFP, fluorescent protein-tagged septins and Lifeact-mCherry began to assemble as a ring between the sister nuclei, suggesting the septin-actin ring formation (Fig. 4h–k). Then, the diameter of the rings became narrower, indicating contraction of the rings (Figs. 4e–g,k–m, 5d,e,i,j). The contraction rates were 0.104 μm/min on average; max: 0.125 μm/min at 30 °C (Supplementary Movie 12). Thus, septum formation was accomplished by the contractile ring containing the septin and actin.

### Subapical peg formation

When the tip of the clamp cell is attached to the subapical cell, CcCla4-EGFP signals emerge at the site where the clamp cell fuses to the subapical hypha, called “subapical peg”^38,41^. The attachment of the clamp tip to the subapical cell appeared to induce the assembly of CcCla4 at the side of the subapical hypha. The CcCla4 assembly in the subapical hypha occurred after the beginning of the contraction of the septin-actin ring at the septum site (Fig. 5d,e; Supplementary Movie 13). When the CcCla4-EGFP signals emerged at the subapical peg, mCherry-CcCdc12 was also assembled at the peg region (Fig. 5e; Supplementary Movie 13). EGFP-CcCdc11a and mCherry-CcCdc3 signals also assembled at the subapical peg region (Fig. 4g). These assemblies suggested that polarized growth occurred to form the subapical peg in the subapical cell.

### Branching

After clamp formation, branching occurred behind the clamp connection (Supplementary Movies 1–3, 7, 9, and 13). At the curved basal part of the branch hypha, fluorescent protein-tagged septins accumulated (Supplementary Movies 1–3), consistent with septin assembly on micron-scale curved membranes. CcCla4-EGFP was assembled at the tip of the branch hypha. Thus, the protrusion of the branch hypha appeared to resemble that of the clamp cell, although the direction of the protrusion was opposite, suggesting a similar molecular mechanism for such protrusion.

## Discussion

**Figure 6:**
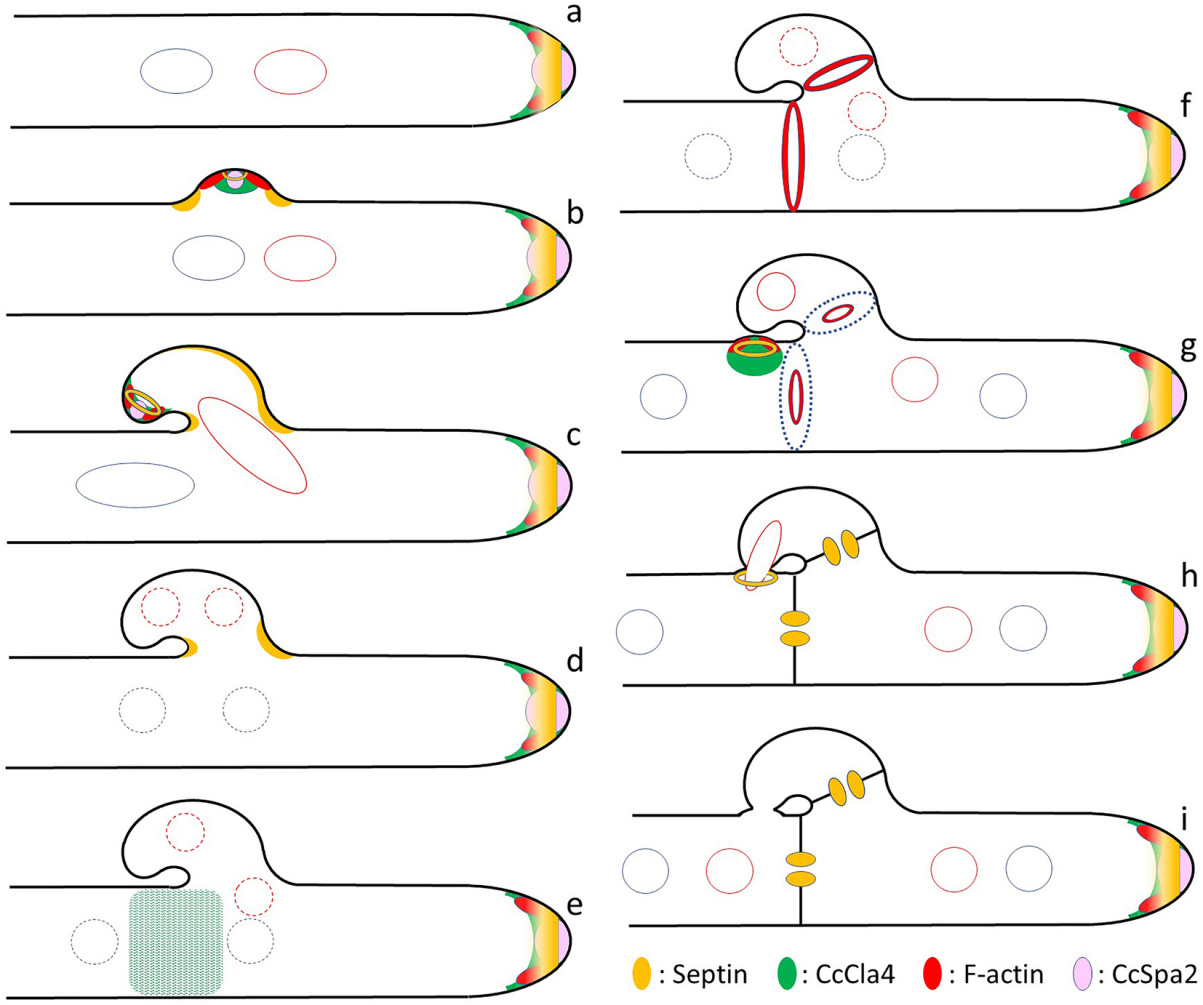
Schematic diagrams showing subcellular localization of septins, CcCla4, F-actin, and CcSpa2 in the growing dikaryotic vegetative hypha of *C. cinerea*. (**a**) The hyphal tip of *C. cinerea* has a tip growth apparatus, which contains a CcCla4 crescent dome, a septin DwH, a polarisome, and fluctuated actin patches. In this diagram, the hyphal tip is depicted not to elongate, but actually elongates. The polarisome contains F-actin, which is not depicted. The two compatible nuclei are depicted by red and blue circles and shown by dashed circles when the nuclear envelope appears to open. (**b**) The protrusion occurs to form the clamp cell. At the tip of the protrusion, another tip growth apparatus emerges. (**c**) The clamp cell grows backward, and the leading nucleus enters the growing clamp cell. (**d**) The conjugate nuclear division occurs. (**e**) After nuclear division and before septation, CcCla4 occasionally assembles around the future septum site transiently. (**f**) The actin and septin rings emerge at the future septum sites. (**g**) The subapical peg is formed in the subapical cell. (**h**) The clamp cell fuses to the peg formed in the subapical cell to release the nucleus trapped in the clamp cell. (**i**) In the new apical cell, the order of the two nuclei derived from (**a**) is exchanged.

Tagging proteins of interest with dual color fluorescent proteins and live cell imaging enabled the examination of the dynamics and relationships between septins and other cell polarity proteins in the growing dikaryotic vegetative hyphae of *C. cinerea*. Different events in the growing dikaryotic vegetative hypha of this model mushroom allow us to dissect and discuss molecular mechanisms by which the fungal cell shape changes in their life cycle. The localizations of Cc-septins, CcCla4, CcSpa2, and F-actin are summarized in Fig. 6.

Assemblies of fluorescent protein-tagged septins at the growing hyphal tip revealed that the septins form a dome with a hole (DwH) at the hyphal tip (Supplementary Movies 1, 2, and 3). EGFP-CcSpa2 visualized the polarisome at the growing hyphal tip (Fig. 3a, Supplementary Movie 9). CcCla4-EGFP signals were observed as a fluctuating dome at the hyphal tip and appeared to be overlapped with both the polarisome and the septin DwH. F-actin visualized by Lifeact-mCherry was also overlapped with the polarisome and the septin DwH. It has been reported that in *S. cerevisiae,* Cla4 kinase phosphorylates certain septins to form septin collar^24^ and a *cla4* mutant is synthetically lethal with a variety of genes, including the genes encoding the polarisome components: Spa2, Pea2, Bni1 and Bud6^25^. The *spa2* mutant is also synthetically lethal with a septin mutant, *cdc10-10*^57^. These findings in *S. cerevisiae* suggest that CcCla4 also exerts influences on both septins and the polarisome at the hyphal tip of the dikaryotic vegetative hypha. In *Bipolaris maydis*, deletion of *cla4* showed hyper-blanching and swelling hyphae^31^. In *A. gossypii*, AgCla4-EGFP localized at the hyphal tip^30^. These findings also suggest a role of Cla4 at the hyphal tip. In *S. cerevisiae*, Hof1 links septins to actin^58^. In future study, it will be interesting to examine whether Hof1 homologs (CC1G_06830, CC1G_04571) localize at the hyphal tip region to link septins to actin.

At the hole of the septin dome at the hyphal tip, there must be the Spitzenkörper, originally described in two *Coprinus* species^59^ and is thought to function as a vesicle supply center to promote hyphal tip growth^60–65^. The Spitzenring of GS-1, which is involved in the synthesis of β-1,3-glucan, has also been reported at the hyphal tip in *N. crassa*^65^. It has been reported in *S. cerevisiae* that when the bud emerges, interplay of Cdc42, septins, and exocysts occurs to confine the Cdc42 activity and exocytosis at the growing bud site^66^. The situation that the septin ring encloses the exocytic region resembles the septin localization observed at the hyphal tip of *C. cinerea*. In *Ustilago maydis*, septin function has been implicated in exocytosis using septin-deletion mutants^19^. The Spitzenkörper also colocalizes with the polarisome and has been discussed in relation to polarisome function^67^. The polarisome has been recognized in *S. cerevisiae* and observed in a variety of filamentous fungi^50,53,67,68^. The localization of *C. cinerea* septins suggested that the septins function at the periphery of the Spitzenkörper and polarisome.

CcCla4-EGFP signals were also observed at the hyphal tip. The region where CcCla4-EGFP signals emerged appeared to be broader than the diameter of the hole of the septin DwH and the polarisome. In addition, the dome of the CcCla4-EGFP signals fluctuated much more than the septin DwH and the polarisome. Similar fluctuation was also observed in actin patches localized at the subapical region. At the subapical region of the hypha, the subapical endocytic ring has been reported in *A. nidulans*^46^, and endocytosis at the hyphal tip has been considered to be an essential process for tip growth^69^. Fluctuated actin patches at the subapical region might be relevant to the subapical endocytic ring. The relationships between endosomes and septin have been investigated in *U. maydis*^70^. In the hyphal tip of *C. cinerea*, septins appeared to exist at the place where both endocytosis and exocytosis should be balanced. Thus, in the subapical region behind the hyphal tip, the relationships between relatively stable Cc-septin DwH and the polarisome, fluctuating CcCla4 and actin patches, and endosomes remain to be elucidated in terms of endocytosis and exocytosis.

As the first indication of clamp cell formation, CcCla4 appeared to assemble at the plasma membrane between two compatible nuclei (Figs. 5a, 6b). This CcCla4 assembly might result from the change in lipid composition of the plasma membrane, which received a signal from the nucleus that decided to start mitosis because it has been reported that the PH domain of Cla4 in *S. cerevisiae* can interact with specific lipids^27^. Then, cortical septins might be accumulated around the CcCla4 assembly site at the clamp cell protrusion. CcSpa2-EGFP and F-actin assembled at the tip of the clamp cell protrusion, resulting in the outgrowth of the clamp cell from the preexisting cell wall. The mechanism by which the clamp cell grows backward to the subapical cell would depend on the position of the anterior nucleus, from which microtubules might elongate and enter the protruding clamp cell^39^. Since CcSpa2-EGFP signals emerged at the tip of the growing clamp cell, the Spitzenkörper and polarisome might form at the tip of the growing clamp cell. While the polarisome stayed at the tip of the clamp cell, the tip might be able to grow continuously (Fig. 6c).

During clamp cell formation, localizations of the Cc-septins dramatically change. The emergence of the protrusion of the clamp cell brings about the plasma membranes with both positive and negative curvatures. The septin DwH in the tip growth apparatus shows the positive curvature of the plasma membrane. The negative curvature emerges at the base of the protrusion, and the septins accumulate at the base of the growing clamp cell. Immediately after nuclear division, the Cc-septins staying on the membrane with positive and negative curvatures are sequestered from the plasma membrane and reassembled as the rings for septation. Septin filaments can sense micrometer-scale membrane curvature^71,72^, and consistent with this, septins are enriched at the curved bases of clamp cells. The cortical septin filaments on the plasma membrane of growing cell regions might enable septin filaments to monitor the membrane curvature all over the cell surface continuously. Further study will be needed to understand dynamic changes in localization of the Cc-septins during clamp cell formation.

When mitosis started, both sumo proteins, CcSumo1 and CcSumo2, disappeared from the nucleus (Fig. 3a; Supplementary Movies 9 and 10), as described in *A. nidulans*^55^. Although some of septins in *S. cerevisiae* have been reported to be sumoylated^36,37^, the *C. cinerea* two sumo proteins, CcSumo1 and CcSumo2, did not show obvious colocalization with Cc-septins. In *Candida albicans*, septins have been reported not to be sumoylated^73^. The proteins that are abundant in cytoplasm and do not enter the nucleus, such as septins, allow us to recognize the nuclear shape as a dark area in cytoplasm (Supplementary Movies 1–3). The dark area disappeared during mitosis, suggesting that the nuclear envelope opened. It is possible that signals to prepare the following events are released from the nucleus by opening the nuclear envelope. Various types of nuclear envelope behaviors in mitosis have been observed in fungal cells: from open mitosis to closed mitosis^38,74,75^. Further study will be required to determine whether “open mitosis” actually occurs in *C. cinerea*.

Following the nuclear division, the actin and septin rings emerged at the septum site. The diameters of these rings together became concurrently narrow (Supplementary Movie 12), indicating contraction of these rings. In *Ashbya gossypii*, when the septum forms, Sep7, Hof1, actin, Myo1, Bud3, and Cyk1 colocalized at the sites of septation^76^. In *S. cerevisiae*, Hof1 interacts with Chs4, which is an activator for the catalytic subunit of chitin synthase-III^77,78^. In experiments using *S. cerevisiae* actin and septins in vitro, Hof1 can physically link actin and septin filaments^58^. The colocalization of septins and actin at the inner edge of the growing septum in our observations, combined with the results in other fungi, suggest collaborative processes of septins and actin in cell wall synthesis during septation. As animal septins are also assembled into the contractile actomyosin ring during cytokinesis^79^, septin functions in cytokinesis may, at least in part, be conserved among fungal and animal cells.

CcCla4 did not assemble at the septum, despite the septin ring forming and contracting at the septum site (Fig. 5; Supplementary Movies 13 and 14), consistent with the observations in *Ashbya gossypii*^30^. However, CcCla4-EGFP signals were occasionally observed around the future septum region after clamp cell formation and before septum formation (Fig. 5g; Supplementary Movie 14). This CcCla4 assembly might be interpreted as preparation for septum formation, because *cla4Δ A. gossypii* mutants lose the ability for septum assembly^30^. This interpretation appears to be consistent with one possible explanation in *A. gossypii* that AgCla4 acts early in establishing the future site of septation^30^. Alternatively, it might be relevant to mitosis, because Cla4 has been identified as a protein encoded by the gene responsible for synthetic lethality with cyclin deletion in *S. cerevisiae*^80,81^. CcCla4 contains a PH domain, which has been reported to interact with specific lipids^27^. The broad assembly of CcCla4 around the future septum site might be related to the emergence of such lipids in the plasma membrane.

In the subapical cell, the subapical peg protrudes from the site near the septum^41^. After clamp cell formation, CcCla4 and Cc-septins assembled at the subapical peg region (Figs. 5e, 6g; Supplementary Movie 13). These assemblies would mean polarized growth to form the subapical peg, as described in *S. commune*^38^. Fusion of the clamp cell to the subapical cell in the mating reaction is regulated by *B*-mating factors, which encode pheromones and pheromone receptors^82,83^. Therefore, the response to form the subapical peg in the subapical cell likely occurs downstream of the pathway controlled by *B*-mating factors^41,84,85^.

*C. cinerea* has two Cdc11 orthologs, CcCdc11a and 11b. Expression of *Cccdc11b* is up-regulated in fruiting, compared to that of *Cccdc11a*^7^. Live cell imaging of CcCdc11b showed that this septin can colocalize with other septins at the hyphal tip, the septum and the basal parts of the protrusion of clamp cells and branching, suggesting that CcCdc11b can form septin complexes with other septins. However, in cytoplasm, CcCdc11b showed a different localization from other septins. The localization suggests that CcCdc11b can associate with tubular structures, such as mitochondria or the endoplasmic reticulum, in the posterior region of the apical cell. In some organisms, septins have been observed at mitochondria^86,87^, which notably present positive membrane curvature. It is possible that CcCdc11b can associate with mitochondria in certain situations. Recent work in *A. gossypii* shows that different Cdc11 variants lead to filaments with different average lengths^88^, potentially consistent with the different dome sizes of CcCdc11a and CcCdc11b. Since Cdc11 occupies the terminal subunit of the septin heterooligomer, it also may have the capacity to control filament flexibility in a manner that may impact the septin assembly on intracellular organelle membranes. Further studies will be required to determine the relationship between organelles and tubular structures associated with septins in the cytoplasm.

In this study, dynamics of *C. cinerea* homologs of players identified in cellular morphogenesis of *S. cerevisiae* were observed in the dikaryotic vegetative hyphae. This fungus can produce fruiting bodies consisting of the obvious cap and stipe, like edible mushrooms, such as *Lentinus edodes* and *Tricholoma matsutake*. Some kinds of cells in fruiting bodies, such as veil cells and stipe cells, show diffuse extension growth, characterized by expansion all over the cell surface^6,89,90^, in contrast to hyphal tip growth in the vegetative hyphae. However, some components of the molecular machinery underlying vegetative hyphal growth should be utilized in fruiting body morphogenesis, as demonstrated in stipe cell elongation^7^. How modification of the vegetative hyphal growth machinery occurs to produce fruiting bodies is still mysterious, and further studies would be needed to unravel the relationship between hyphal tip growth and diffuse extension growth in fruiting body morphogenesis of this mushroom. Since edible mushrooms should share in part the molecular mechanisms for fruiting in *C. cinerea*, identification and regulation of key steps in the transition from vegetative hyphal growth to diffuse extension growth would improve the edible mushroom cultivation conditions.

## Methods

### Strains, culture conditions and cloning

The strains used are listed in Supplementary Table S1. The mycelia were grown on MYG (1% malt extract, 0.4% yeast extract, 0.4% glucose, 1.5% agar) medium for usual culture, or in TM (2% glucose, 10 mM (NH_4_)_2_HPO_4_, 0.12 µg/ml thiamine, 0.05% MgSO_4_·7H_2_O, 10 mM potassium phosphate, pH7.0, in autoclaved tap water) liquid medium for observations of the growing vegetative hyphae.

Twelve proteins were tagged with EGFP, PA-GFP and/or mCherry, as described in Supplementary Table S2. Primers, plasmids, and cloning methods used to construct expression vectors are listed in Supplementary Table S3. For CcCdc3 tagging see our previous report^7^. Gene sawing PCR and FastCloning^91^ were mainly used for cloning. pLifeact-mCherry-7 was purchased from Addgene and driven by actin promoter and terminator.

### Transformation

The protoplasts of strain #292 (*A3B1 trp1-1, 1–6*) or #8 (*A2B2 trp1-1, 1–6*) were transformed with a mixture of pCc1003 (*trp1*^+^) and the constructed expression vectors, as described previously^92^. The trp^+^ transformants were recovered on minimal medium to purify the transformed cells. The purified trp^+^ transformants were observed by a fluorescence microscope to check expression of tagged proteins and selected for mating to construct dikaryons. Transformation in *C. cinerea* results in the random insertion of the expression vectors into the genome, causing the situation where both native and tagged proteins are expressed in a cell.

### Microscopy and image processing

A small agar (1 mm cube) with hyphae was inoculated in 100–200 µl of a liquid TM medium on a coverslip and incubated at 28–30 °C for 16–24 h. After removing the liquid medium, the inverted coverslip with the hyphae was placed on 4% agarose pad, in which a hole was made and filled with the removed medium from the coverslip to set the inoculum. After 1 h or more, the vegetative hyphae were observed by fluorescence microscopes. The fluorescence microscopes used and image capturing conditions are listed in Supplementary Table S4. Arduino was used for exchanging filters and opening shutters when the microscope used did not equip such functions, as described^93^. Images were processed with ImageJ and/or iMovie and exported as MOV files by PowerPoint. The MOV files were compressed by VideoSmaller (https://www.videosmaller.com/) to less than 10 Mb.

## Supplementary Information

Supplementary Video 1.

Supplementary Video 2.

Supplementary Video 3.

Supplementary Video 4.

Supplementary Video 5.

Supplementary Video 6.

Supplementary Video 7.

Supplementary Video 8.

Supplementary Video 9.

Supplementary Video 10.

Supplementary Video 11.

Supplementary Video 12.

Supplementary Video 13.

Supplementary Video 14.

Supplementary Information.

## Data Availability

The datasets used and/or analysed during the current study are available from the corresponding author upon reasonable request. All data generated or analysed during this study are included in this published article [and its supplementary information files].
