# Supplementary material for: Live-cell imaging of septins and cell polarity proteins in the growing dikaryotic vegetative hypha of the model mushroom Coprinopsis cinerea
Source: Sci Rep. 2023 Jun 22;13:10132. doi: 10.1038/s41598-023-37115-y (PMC10287680; doi:10.1038/s41598-023-37115-y)
Supplement: Supplementary file 15 — Supplementary Information. [file 41598_2023_37115_MOESM15_ESM.pdf]

Supplemental information - Live-cell imaging of septins and cell polarity proteins in the growing dikaryotic vegetative hypha of the model mushroom *Coprinopsis cinerea*

Tetsuya Kakizaki[1], Haruki Abe[1], Yuuka Kotouge[1], Mitsuki Matsubuchi[1], Mayu Sugou[1], Chiharu Honma[1], Kouki Tsukuta[1], Souichi Satoh[1], Tatsuhiro Shioya[1], Hiroe Nakamura[1], Kevin S. Cannon[2], Benjamin L. Woods[2], Amy Gladfelter[2], Norio Takeshita[3], Hajime Muraguchi[1,*]

[1]Department of Biotechnology, Faculty of Bioresource Sciences, Akita Prefectural University, Akita 010-0195, Japan

[2]Department of Biology, University of North Carolina at Chapel Hill, NC, USA

[3]School of Life and Environmental Sciences, University of Tsukuba, Ten-Nou-Dai, Tsukuba 305-8572, Japan

Supplementary information

Movie list

Movie 1: EGFP-CcCdc11b and mCherry-CcCdc3 in the apical cell
Movie 2: EGFP-CcCdc10 and mCherry-CcCdc12 in the apical cell
Movie 3: EGFP-CcCdc11a and mCherry-CcCdc3 in the apical cell
Movie 4: 3D image of EGFP-CcCdc10 in the apical cell
Movie 5: PA-GFP-CcCdc3 in the apical cell
Movie 6: CcCla4-EGFP and mCherry-CcCdc3 in the apical cell
Movie 7: CcCla4-EGFP and mCherry-CcCdc12
Movie 8: EGFP-CcCdc10 and Lifeact-mCherry at the hyphal tip
Movie 9: CcSpa2-EGFP and mCherry-CcSumo1 in the growing hypha
Movie 10: Histone H1-EGFP and mCherry-CcSumo2 in conjugate nuclear division
Movie 11: Histone H1-EGFP
Movie 12: EGFP-CcCdc10 and Lifeact-mCherry in the apical cell
Movie 13: CcCla4-EGFP and mCherry-CcCdc12 in clamp formation
Movie 14: CcCla4-EGFP and mCherry-CcCdc3 during septation

Table S1. Strains used.

Strain	Genotype / Description	Source / Reference
Okayama-7	*A2 B2 ade8-1*	Moore et al., 1979
#292	*A3 B1 trp1-1, 1-6*	P. J. Pukkila
#8	*A2 B2 trp1-1, 1-6* / F1 progeny between Okayama-7 and #292	This study
B87	*A12 B1 trp1-1, 1-6 CcCdc3-1*	Shioya et al., 2013
#292'#10	*A3 B1 Histone H1-EGFP*	This study
Dik#1	*A12 B1 EGFP-CcCdc11a + A2 B2 mCherry-CcCdc3* / B87-transformant #4 × #8-transformant #5	This study
Dik#5	*A12 B1 EGFP-CcCdc11b + A2 B2 mCherry-CcCdc3* / B87-transformant #8 × #8-transformant #8	This study
Dik#9	*A12 B1 EGFP-CcCdc10 + A2 B2 mCherry-CcCdc12* / B87-transformant #1 × #8-transformant #2	This study
Dik#13	*A3 B1 CcCla4-EGFP + A2 B2 mCherry-CcCdc3* / #292-transformant #1 × #8-transformant #5	This study
Dik#15	*A3 B1 CcCla4-EGFP + A2 B2 mCherry-CcCdc12* / #292-transformant #1 × #8-transformant #2	This study
Dik#20	*A2 B2 EGFP-CcCdc10 + A3 B1 Lifeact-mCherry* / Okayama-7F1#1 × #292-transformant #2	This study
Dik#24	*A3 B1 Histone H1-EGFP + A2 B2 mCherry-CcSumo2* / #292-transformant #2 × #8-transformant #3	This study
Dik#33	*A3 B1 CcSpa2-EGFP + A2 B2 mCherry-CcSumo1* / #292-transformant #31 × #8-transformant #2	This study
Dik#39	*A3 B1 Histone H1-EGFP + A2 B2 ade8-1* / #292-transformant #10 × Okayama-7	This study
Dik#101	*A3 B1 PA-GFP-CcCdc3 + A2 B2 ade8-1* / #292-transformant #11 × Okayama-7	This study

Table S2. Tagged proteins

	Protein tagged	CC1G_No.	No. of amino acids	Tagged terminus	Fluorescent tag		
1	CcCdc3	CC1G_10270	441	N	EGFP	mCherry	PA-GFP
2	CcCdc10	CC1G_12292	313	N	EGFP		
3	CcCdc11a	CC1G_03701	367	N	EGFP		
4	CcCdc11b	CC1G_03218	363	N	EGFP		
5	CcCdc12	CC1G_02638	383	N	EGFP	mCherry	
6	CcCla4	CC1G_02087	810	C	EGFP		
7	CcSpa2	CC1G_02018	924	C	EGFP		
8	CcSumo1	CC1G_04810	100	N		mCherry	
9	CcSumo2	CC1G_15791	94	N		mCherry	
10	Histone CcH1	CC1G_03813	313	C	EGFP		
11	Lifeact		17	C		mCherry	

Table S3. Primers and cloning method

Target, promoter and terminator	Primer name	Primer sequence	Used Vector and cloning method
CcCdc10 used the native promoter and terminator	1-PstI-Cdc10(P)-For<br>2-EGFP-Cdc10(P)-Rev<br>3-Cdc10(P)-EGFP-For<br>4-Cdc10-EGFP-Rev<br>5-EGFP-Cdc10-For<br>6-SalI-Cdc10(T)-Rev	GCGCCTGCAGTCTTCCTCTTCTTCACTCC<br>CTCCTCGCCCTTGCTCACCATTGTTGGGTTGGCAGACGATGG<br>CCATCGTCTGCCAACCCAACAATGGTGAGCAAGGGCGAGGAG<br>GCGGATTTCTTCAGTCATCTTGTACAGCTCGTCCAT<br>ATGGACGAGCTGTACAAGATGACTGAAGAAATCCGC<br>GCGCGTCGACTTCGTCCGCTTCGTGGTGG	pBluescript II SK by Gene sewing PCR, digestion with RE and ligation
CcCdc11a used the native promoter and terminator	1-pEGFP-Cdc11a(P)-For<br>2-EGFP-Cdc11a(P)-Rev<br>3-EGFP-Cdc11a(T)-For<br>4-pEGFP-Cdc11a(T)-Rev	CTGCAGTCGACGGTACCGAATAGAACTCTCCGGTC<br>CTCGCCCTTGCTCACCATAGCTGCTTGAATTTGTGG<br>ATGGACGAGCTGTACAAGATGTCGTTCGCAGGAAGA<br>ATCTAGAGTCGCGGCCGCAGTAGACTGGTCGACCAC	pEGFP-1 by FastCloing
CcCdc11b used the native promoter and terminator	1-KpnI-Cdc11b(P)-For<br>2-EGFP-Cdc11b(P)-Rev<br>3-Cdc11b(P)-EGFP-For<br>4-Cdc11b-EGFP-Rev<br>5-EGFP-Cdc11b-Ror<br>6-ApaI-Cdc11b(T)-Rev	GCGGTACCCGGCATATTTGGCAAGTGAGC<br>CTCGCCCTTGCTCACCATTTTTGTGGGTGGGCAAGG<br>CCTTGCCCACCCACAAAAATGGTGAGCAAGGGCGAG<br>GTCGAGCAGGGAGCGGCATCTTGTACAGCTCGTCCAT<br>ATGGACGAGCTGTACAAGATGCCGCTCCCTGCTCGAC<br>GCGGGCCCCACCTGTGATTACCACAACC	pBluescript II SK by Gene sewing PCR, digestion with RE and ligation
CcCdc12 used the native promoter and terminator	VpEGFP1-KpnI-Rev<br>1-pEGFP-Cdc12(P)-For<br>2-EGFP-CcCdc12(P)-Rev<br>3-EGFP-Cdc12-(T)-For<br>4-pEGFP-Cdc12(T)-Rev<br>VpEGFP-NotI-For	GTACCGTCGACTGCAG<br>CTGCAGTCGACGGTACCATCCATCGTGGGCTGGG<br>CTCGCCCTTGCTCACCATCCTGGAACTGGCTGTGGG<br>ATGGACGAGCTGTACAAGATGTCTGCTCTCTCGACT<br>GATCTAGAGTCGCGGCCGCACCCCGACGACACGTTCC<br>CGGCCGCGACTCTAGATC	pEGFP-1 by FastCloing
CcCla4 used the native promoter and terminator	1-PstICla4(P)-For<br>2-(P)Cla4-PstI-Rev<br>3-XbaI-Cla4(T)-For<br>4-XbaI-Cla4(T)-Rev	GCGCCTGCAGCGCCACCCCCAGAGCGGGTG<br>GCGCCTGCAGGGACGCTTGCTTGGTCTTGAAG<br>GCGCTCTAGAACGCTTGTGTTGGTCGTTTATG<br>GCGCTCTAGAACTGGACAAGACGCCGTC	pEGFP-1, pBluescript II SK pUC118 by PCR, digestion with RE and ligation
CcSpa2 used the native promoter and terminator	1-SacI(P)Spa2-For<br>2-EGFP-KpnI-Rev<br>3-NotI-Spa2(T)-For<br>4-XbaI-Spa2(T)-Rev	GCGCGAGCTCACCAAGGCGTTCCTGTCCAC<br>GCGCGGTACCAACTTCATGAGACCCTTCAT<br>GCGCGCGGCCGCTTATGGATTCTTTGGACTCG<br>GCGCTCTAGAGCGATTGGTCCACGGACAAG	pEGFP-1 by PCR, digestion with RE and ligation
CcSumo1 used the native promoter and terminator	1-ApaI-Sumo1(P)-For<br>2-EGFP-Sumo1(P)-Rev<br>3-Sumo1(P)-EGFP-For<br>4-Sumo1-EGFP-Rev<br>5-EGFP-Sumo1-For<br>6-BamHI-Sumo1(T)-Rev	GCGCGGGCCCGCTTCTATGGAGAGAGCGAC<br>CTCGCCCTTGCTCACCATGTTGTACGGTGATTACTAG<br>CTAGTAATCACCGTACAACATGGTGAGCAAGGGCGAG<br>CTGCTCCTCGTCAGACATCTTGTACAGCTCGTCCAT<br>ATGGACGAGCTGTACAAGATGTCTGACGAGGAGCAG<br>GCGCGGATCCATGAGGTGCCAGTAGGTC	pBluescript II SK by Gene sewing PCR, digestion with RE and ligation
CcSumo2 used the native promoter and terminator	gCcSumo2-For<br>gCcSumo2-Rev<br>Sumo2(P)ATG(FP)-Rev<br>(FP)Sumo2ATG-For	AAATCCACGATCCCCGTTGGC<br>CATCGCGGACGGTAGTCGTTC<br>GCCCTTGCTCACCATCGTTGGATTTCCTATTCTTC<br>GACGAGCTGTACAAGATGAGCCAGGAACCTGAG	pGEM-T Easy (Promega) by TA-cloning and FastCloning
Histone H1 used the native promoter and terminator	1-CcH1(P)-For<br>2c-H1-EGFP-Rev<br>3c-EGFP-H1(T)-For<br>4-H1(T)-Vp-Rev	CTGCAGTCGACGGTACGCTACCGTTTGCGACATG<br>CTGGCCCTTGCTCACCATGGCACTGGTAGTGGCAGC<br>ATGGACGAGCTGTACAAGTAAATGGATATGTACCTTC<br>GATCTAGAGTCGCGGCCGTGTTGGGAAGACGTGTTG	pEGFP-1 by FastCloing
Lifeact used the Actin (CC1G_08232) promoter and terminator	VpLifeact-Rev<br>Lifeact-ATG-For<br>1-vpLA_Actin(P)-For<br>2-Actin(P)_LA-Rev<br>3-mChe_Actin(T)-Rev<br>4-Actin(T)-Rev	CGGAACTCCATATATGGG<br>ATGGGCGTGGCCGACTTG<br>CCCATATATGGAGTTCCGAGCAGAGTCCTAGAACCAG<br>CAAGTCGGCCACGCCCATGGTGATTATTCGTTAAGTG<br>ATGGACGAGCTGTACAAGTAAACGACCTCCTTACGAC<br>GATCTAGAGTCGCGGCCGCTGACCGACTGAGCGAGAG	pmCherry-Lifeact-7 (Addgene) by FastCloing
EGFP, PA-GFP or mCherry	EGFP-ATG-For<br>EGFP-AAG-Rev<br>out_EGFP-CAT-Rev<br>out_EGFP-AAG-For	ATGGTGAGCAAGGGCGAG<br>CTTGTACAGCTCGTCCAT<br>CTCGCCCTTGCTCACCAT<br>ATGGACGAGCTGTACAAG	FastCloning for replacing EGFP with mCherry or PA-GFP

Table S4. Image capturing conditions.

			Fluorescent tag						
Movie No.	Figure	Strain	EGFP	mCherry	PA-GFP	Microscope	Camera	Software	Additional Equipment
Movie 1	Fig. 1 a	Dik#5	-CcCdc11b	-CcCdc3	-	Ti (Nikon)	Prime 95B	NIS-Elements	Spinning Disk: CSU-W1
Movie 2	Fig. 1 c	Dik#9	-CcCdc10	-CcCdc12	-	Ti (Nikon)	Prime 95B	NIS-Elements	Spinning Disk: CSU-W1
Movie 3	Fig. 1 e,<br>Fig. 4 a-g	Dik#1	-CcCdc11a	-CcCdc3	-	Ti (Nikon)	Prime 95B	NIS-Elements	Spinning Disk: CSU-W1
Movie 4	Fig. 1 g	Dik#9	-CcCdc10	-	-	AX R (Nikon)	-	NIS-Elements	-
Movie 5	Fig. 1 h, i	Dik#101	-	-	-CcCdc3	AX R (Nikon)	-	NIS-Elements	Lens heater (Tokai Hit)
Movie 6	Fig. 2 a	Dik#13	CcCla4-	-CcCdc3	-	Axio Observer Z1<br>(Carl Zeiss)	AxioCam 506	ZEN Software<br>(Version 3.5, Carl Zeiss)	Thermo Plate (Tokai Hit)
Movie 7		Dik#15	CcCla4-	-CcCdc12	-	Ti (Nikon)	Prime 95B	NIS-Elements	Spinning Disk: CSU-W1
Movie 8	Fig. 2 c	Dik#20	-CcCdc10	Lifeact-	-	Axio Observer Z1<br>(Carl Zeiss)	AxioCam 506	ZEN Software<br>(Version 3.5, Carl Zeiss)	Thermo Plate (Tokai Hit)
Movie 9	Fig. 3 a	Dik#33	CcSpa2-	-Sumo1	-	Ti (Nikon)	Prime 95B	NIS-Elements	Spinning Disk: CSU-W1
Movie 10		Dik#24	Histone H1-	-Sumo2	-	E600 (Nikon)	DP72 (Olympus)	Lumina Vision<br>(MITANI CORPORATION)	Arduino for filter change
Movie 11	Fig. 3 b, c	292'#10×Ok7	Histone H1-	-	-	E600 (Nikon)	DP72 (Olympus)	Lumina Vision<br>(MITANI CORPORATION)	Arduino for filter change
Movie 12	Fig. 4 h-m	Dik#20	-CcCdc10	Lifeact-	-	Axio Observer Z1<br>(Carl Zeiss)	AxioCam 506	ZEN Software<br>(Version 3.5, Carl Zeiss)	Thermo Plate (Tokai Hit)
Movie 13	Fig. 5 a-e	Dik#15	CcCla4-	-CcCdc12	-	Ti (Nikon)	Prime 95B	NIS-Elements	Spinning Disk: CSU-W1
Movie 14		Dik#13	CcCla4-	-CcCdc3	-	Ti (Nikon)	Prime 95B	NIS-Elements	Spinning Disk: CSU-W1

	Excitation				Exposure time				Time lapse		
Movie No.	Light source	EGFP	mCherry	PA-GFP	Bright field	EGFP	mCherry	PA-GFP	interval	Culture	Temperature
Movie 1	Laser	488	561	-	50 msec	100 msec	200 msec	-	20 sec	on agarose pad	RT
Movie 2	Laser	488	561	-	50 msec	100 msec	200 msec	-	20 sec	on agarose pad	RT
Movie 3	Laser	488	561	-	50 msec	100 msec	200 msec	-	20 sec	on agarose pad	RT
Movie 4	Laser	488	561	-	-	-	-	-		on agarose pad	RT
Movie 5	Laser	-	-	488	-	-	-	-	5 sec	on agarose pad	37℃
Movie 6	Colibri.2 LED light<br>(Carl Zeiss)	488	561	-	-	-	-	-	6 sec	in liquid media	30℃
Movie 7	Laser	488	561	-	50 msec	100 msec	200 msec	-	20 sec	on agarose pad	RT
Movie 8	Colibri.2 LED light<br>(Carl Zeiss)	488	561	-	-	-	-	-	1 sec	in liquid media	30℃
Movie 9	Laser	488	561	-	50 msec	100 msec	200 msec	-	20 sec	on agarose pad	RT
Movie 10	Hg lamp	Filter	Filter	-	-	3 sec	3 sec	-	1 min	on agarose pad	RT
Movie 11	Hg lamp	Filter	Filter	-	1/15 sec	8 sec	-	-	40 sec	on agarose pad	RT
Movie 12	Colibri.2 LED light<br>(Carl Zeiss)	488	561	-	-	-	-	-	5 min	in liquid media	30℃
Movie 13	Laser	488	561	-	50 msec	100 msec	200 msec	-	20 sec	on agarose pad	RT
Movie 14	Laser	488	561	-	50 msec	100 msec	200 msec	-	20 sec	on agarose pad	RT
